# SARS Outbreak in Taiwan

**DOI:** 10.3201/eid1008.040115

**Published:** 2004-08

**Authors:** Po-Ren Hsueh, Pan-Chyr Yang

**Affiliations:** * National Taiwan University College of Medicine, Taipei, Taiwan

**Keywords:** SARS, Taiwan, reply

**To the Editor:** The article by Hsieh et al. analyzed the daily case-report data for severe acute respiratory syndrome (SARS) from May 5 to June 4, 2003, posted on the Web site for the Taiwan Center for Disease Control, to show how this disease had rapidly spread in the 2003 outbreak (*1*). Hsieh et al. suggested that infection in hospitalized patients who were classified erroneously as suspected SARS case-patients was a major factor in the rapid spread of the disease in hospitals. Slow classification and delayed placement of these patients in negative-pressure isolation rooms contributed to the high percentage (73%) of nosocomial infection in Taiwan (*1*).

During the outbreak period (stage II), three teams were responsible for classifying SARS cases (*2*). The team included infectious disease specialists, respiratory specialists, and epidemiologists recruited from major teaching hospitals throughout Taiwan and was organized by the Taiwan Center for Disease Control and the National Health Insurance Bureau. The team met daily and reviewed the clinical data, travel and contact history, and chest radiographic scans of the reported case-patients obtained (by email or fax) from the patients' attending physicians. The same protocol (Figure) was used by all team members to classify the case-patients as having suspected or probable SARS. All hospitals that treated patients with suspected SARS either had their own committee to classify patients according to World Health Organization guidelines or followed the protocol for classification or reclassification of reported cases by the team members (*3*).

**Figure:**
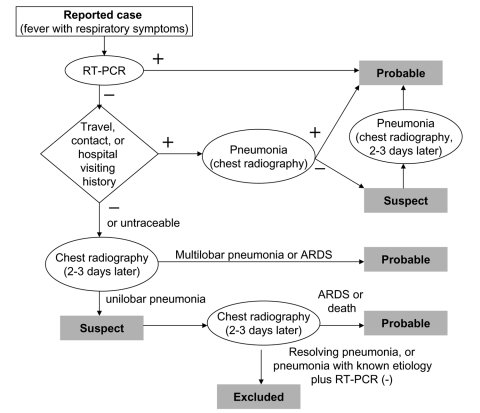
Flowchart of classification for severe acute respiratory syndrome (SARS) revised on May 1, 2003. ARDS, acute respiratory distress syndrome.

Although official reclassification might have taken 12.5 days as suggested by Hsieh et al., the conclusion that inadequate isolation of infected patients during this period led to a higher rate of nosocomial transmission cannot be based on the data available to these authors. From the first day that suspected cases were reported to the Taiwan Center for Disease Control, the patients were placed in negative-pressure isolation rooms when available. Suspected case-patients may have been less likely than probable case-patients to be placed in negative-pressure isolation rooms when these were in short supply; however, all other available isolation precautions were used to treat suspected case-patients before they were reclassified. The notion that increased infection transmission occurred despite these isolation precautions is not consistent with the literature suggesting the central role of gloves, gowns, and surgical masks in preventing transmission (*4*). Thus, the process of reclassification was not associated with the timing of isolation measures shown to have the greatest impact in preventing infection transmission.

The high proportion of patients with nosocomial SARS infection in Taiwan is consistent with the observations of Lingappa et al. (*5*) and others who have noted that the hospital setting was the primary amplifier of SARS transmission, with significant community transmission occurring in only the largest outbreaks. The high proportion of nosocomial cases suggests that containment measures instituted in Taiwan were ultimately successful in preventing a much larger outbreak. Multiple factors were associated with the nosocomial outbreaks in Taiwan, including inadequate infection control infrastructure and triage screening that led to delayed detection of several highly contagious index cases.
