# Supplementary material for: SARS Outbreak in Taiwan
Source: Emerg Infect Dis. 2004 Aug;10(8):1514–5. doi: 10.3201/eid1008.040115 (PMC3320396; doi:10.3201/eid1008.040115)
Supplement: Related texts

# Reply: SARS Outbreak in Taiwan

**DOI:** 10.3201/eid1008.040329

**Authors:** Ying-Hen Hsieh, Cathy W.S. Chen, Sze-Bi Hsu

**Affiliations:** * National Chung Hsing University, Taichung, Taiwan;; † Feng Chia University, Taichung, Taiwan;; ‡ National Tsing Hua University, Hsinchu, Taiwan

**In Reply:** Hsueh and Yang (*1*) correctly described the case classification procedure in Taiwan during the 2003 severe acute respiratory syndrome (SARS) outbreak as being conducted simultaneously by three teams of local experts in the northern, central, and southern parts of Taiwan; however, they failed to mention that this procedure was implemented only after May 10 (*2*). More precisely, before May 9, the relevant medical records of all reported SARS case-patients were reviewed by a SARS Advisory Committee at the Taiwan Center for Disease Control, whose members included respiratory specialists, infectious disease physicians, and epidemiologists. After May 10, because the dramatic increase in the number of new cases attributed to the hospital cluster outbreaks in Taipei in late April (*3*), the SARS Advisory Committee at the Taiwan Center for Disease Control in Taipei could no longer effectively provide swift review for the rapidly increasing case load. Consequently, three regional offices of the Bureau of National Health Insurance (BNHI) north, central, and south of Taiwan took over the responsibility of case review and used standard operating procedures for case evaluation (*2*). Local SARS expert committees were established in all three regions, with each committee consisting of the relevant experts. This policy change provides irrefutable evidence that the authorities expedited the case classification process, which was deemed too slow, because the backlog of cases waiting to be reviewed was mounting.

In a subsequent, related study (Hsieh et al., unpub. data), retrospective statistical analysis of the laboratory–confirmed case data conducted with a two-sample *t* test indicated that the mean time from initial diagnosis of patients with suspected SARS to reclassification as probable SARS, improved significantly after May 10. The estimated mean time from diagnosis to reclassification was 12.56 days from May 5 to June 4 (*3*). Final classification was substantially delayed in the suspected SARS cases that were reclassified as probable SARS cases in the days after the new procedure was implemented. However, they were well represented in our mean estimation result.

Another issue raised by Hsueh and Yang concerns the evidence of nosocomial infections. From May 5 to June 4, the suspected SARS patients in Taiwan were placed in negative pressure chambers, when available, as soon as they were diagnosed. However, the operative word here is "when available." In National Taiwan University Hospital, the most established and well-equipped hospital in Taiwan, swift and efficient isolation was accomplished. Only 31 SARS cases, a small fraction of the Taiwan case data, occurred through exposure in the emergency room at the National Taiwan University Hospital Hospital, which culminated in the temporary shutdown of emergency services on May 12 (*3**,**4*). Other hospitals in Taiwan had cluster infections on wards as late as the end of May (*5**,**6*). Multiple factors were associated with the nosocomial outbreaks in Taiwan. Our modeling result merely suggested that the slow classification process, which was effectively rectified with the policy change on May 10, had been one of the contributing factors, and the change was subsequently instrumental in the quick containment of the outbreak. The intervention efforts helped prevent SARS infection transmission in medical facilities from spreading into the community. Nonetheless, a more proactive and constructive approach is to learn from this experience and to minimize the opportunity for nosocomial infections to occur in the future.

Finally, we stressed that "with more and better data, one could perhaps estimate the parameters over smaller periods of interest during the complete progression of the epidemic, if not the parameter values for each time n" (*3*). In the last 2 decades, the academic literature contains abundant evidence of how mathematical modeling can provide insights into infectious diseases (*7*). The purpose of mathematical modeling is to reconstruct the epidemic events of importance from the data that are available at the time. Our modeling was conducted during the summer before the end of the epidemic and only the data available from various Web sites was used, which did not allow us to compare the difference in classification time before and after May 10. With the laboratory-confirmed SARS case data now available, we are able provide more definitive and detailed results in a manuscript under review (Hsieh et al., unpub. data), as well as in an ongoing modeling of the SARS outbreak in Taiwan that encompasses intervention measures and behavior change of the general public.

With the current void of precise knowledge regarding the chains of infections that led to the in-hospital and inter-hospital infections in Taiwan, mathematical modeling gives the best hope of understanding exactly how the cluster infections occurred, so we can better meet the challenges of future epidemics. Such knowledge is possible only with the interface of detailed epidemiologic and molecular data of the SARS cases with mathematical modeling. During this past winter season, a second wave of the SARS epidemic was averted. Hopefully, retrospective modeling studies such as ours will better prepare us for the emergence of any infectious diseases in the future.

## References

[1] Hsueh PR, Yang PC. SARS outbreak in Taiwan [letter]. Emerg Infect Dis. 2004;10:1514–5. 10.3201/eid1008.04011515503404PMC3320396

[2] Center for Disease Control-Taiwan. SARS major timeline. In: Memoir of Severe Acute Respiratory Syndrome Control in Taiwan. Taipei: Center for Disease Control (Taiwan); 2003. p. 67–81.

[3] Hsieh YH, Chen CWS, Hsu SB. SARS outbreak, Taiwan 2003. Emerg Infect Dis. 2004;10:201–6.1503068310.3201/eid1002.030515PMC3322921

[4] Chen YC, Huang LM, Chan CC, Su CP, Chang SC, Chang YY, SARS in hospital emergency room. Emerg Infect Dis [serial on the Internet]. [cited 2004 Apr 3]. Available from: http://www.cdc.gov/ncidod/EID/vol10no5/03-0579.htm10.3201/eid1005.030579PMC332322315200809

[5] Jiang DD, Wang KF, Su IJ. Incident of clustering infections of SARS in the Kaohsiung Chang Gung Memorial Hospital. In: Memoir of Severe Acute Respiratory Syndrome Control in Taiwan. Taipei: Center for Disease Control (Taiwan); 2003. p. 49–50.

[6] Jiang DD, Peng MM, Wang JN, Lu LH, Chang H, Su IJ. Incident of SARS clustering infections in the Taipei Municipal Yang-Ming Hospital. In: Memoir of Severe Acute Respiratory Syndrome Control in Taiwan. Taipei: Center for Disease Control (Taiwan); 2003. p.51–3.

[7] Anderson RM, May RM. Infectious diseases of human. Oxford: Oxford University Press; 1991.
